# Supplementary material for: Fabrication of EVOH/PANI Composite Nanofibrous Aerogels for the Removal of Dyes and Heavy Metal Ions
Source: Materials (Basel). 2023 Mar 16;16(6):2393. doi: 10.3390/ma16062393 (PMC10054761; doi:10.3390/ma16062393)
Supplement: Supplementary file 1 [file materials-16-02393-s001.zip › materials-2257981-supplementary.pdf]

Supplementary Materials of

Fabrication of EVOH/PANI Composite Nanofibrous Aerogels for Removal of Dyes and Heavy Metal Ions

Junshan Zhu[1]*, Hang Lu [1], Jianan Song[2]*

1. *Jiangsu Petroleum Branch of Sinopec Sales Co., Ltd., Nanjing, 210003, China*

2. *Research School of Polymeric Materials, School of Materials Sciences & Engineering, Jiangsu University, Zhenjiang, 212013, China*

Correspondence to: Junshan Zhu (Email: 13262567603@163.com)

Jianan Song (Email: songjianan@ujs.edu.cn)

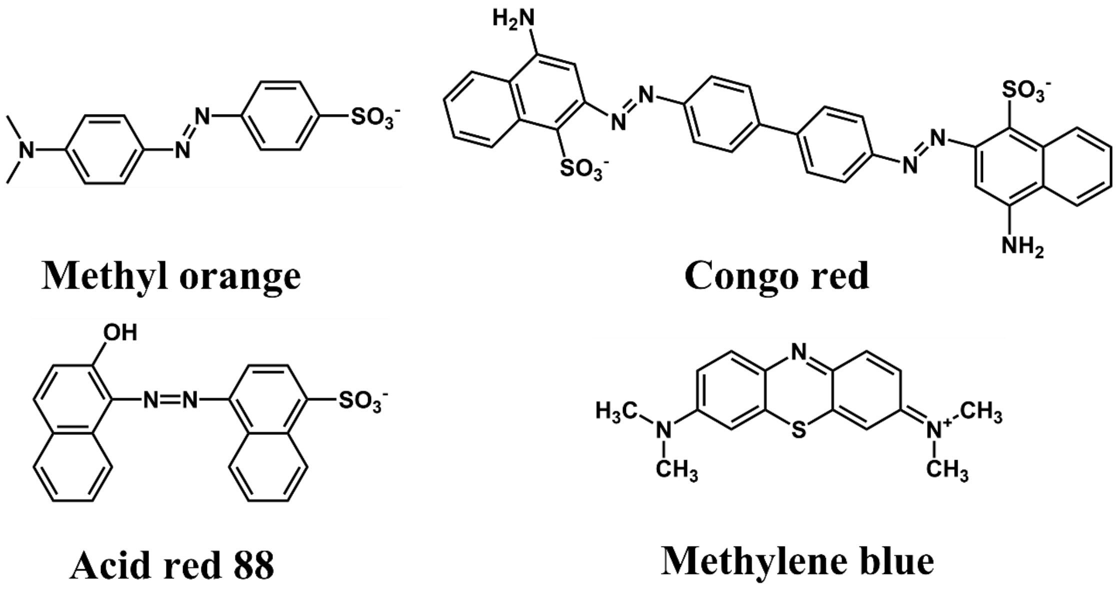

Figure S1 The molecular structure of dyes

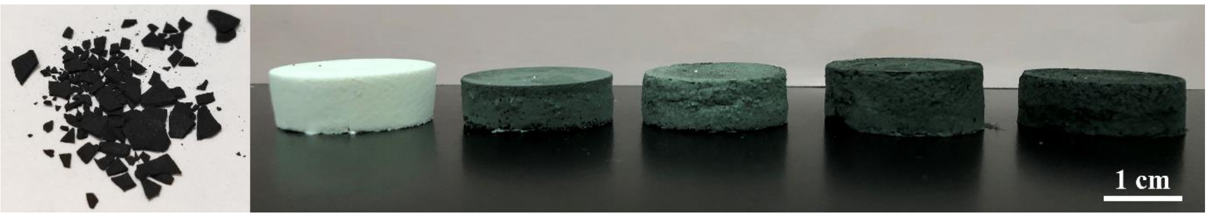

Figure S2 Photograph of EVOH/PANI composite NFAs

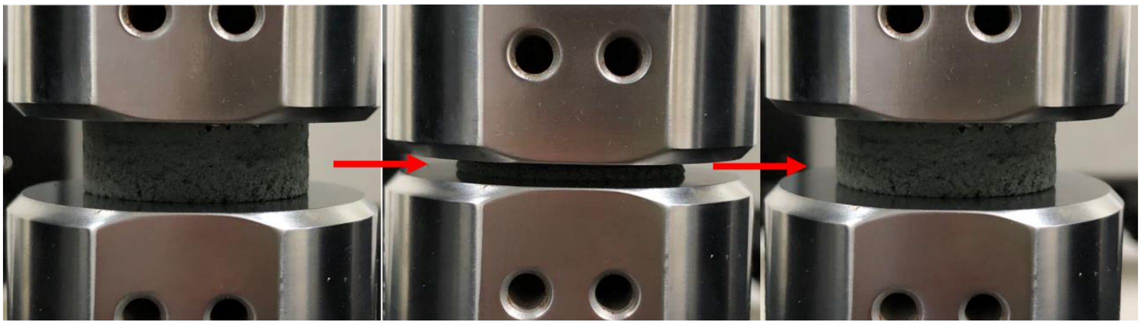

Figure S3 photographs of EP-1 under a compression and release (ε = 80%)

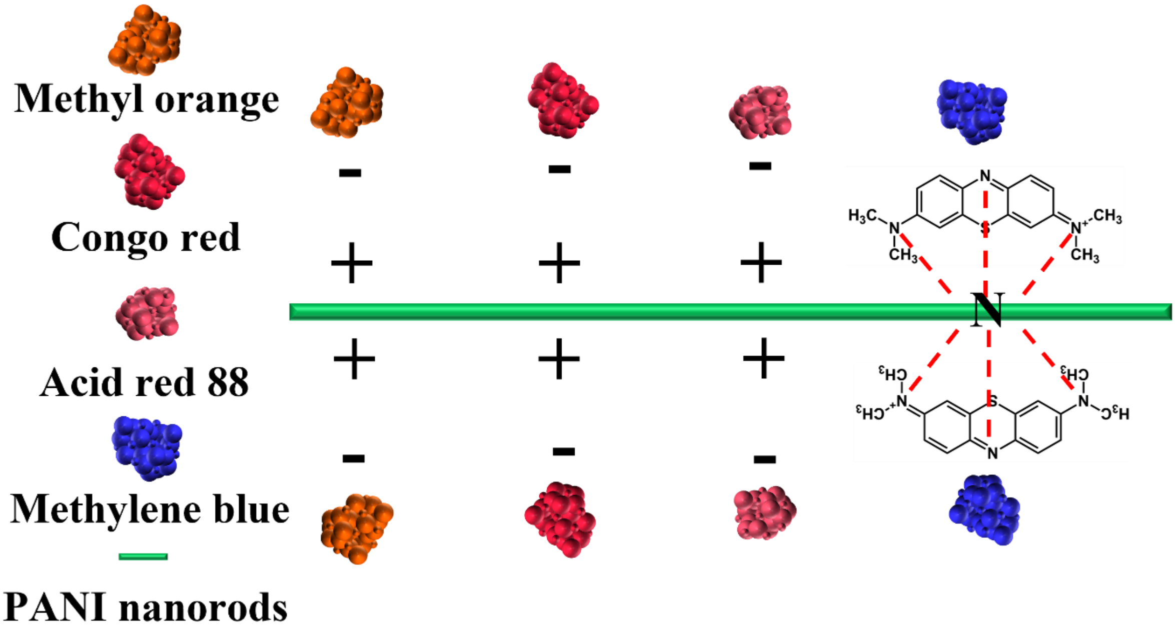

Figure S4 The mechanism of dyes adsorption

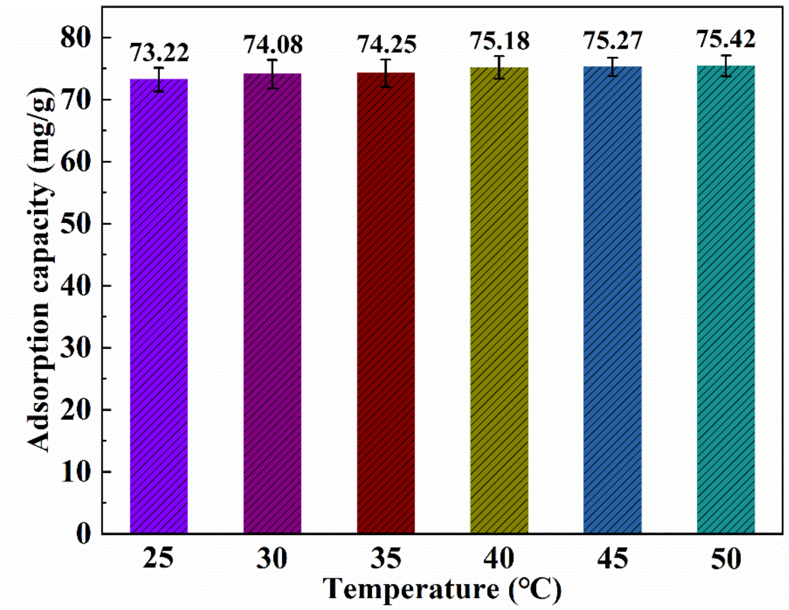

Figure S5 The adsorption capacity of EP-3 for methyl orange under different temperature
